# Relatively Benign Clinical Course of Acute Pneumonitis After Pleurodesis Using *Viscum album* Extract in Very Elderly Patient

**DOI:** 10.1155/crpu/5682658

**Published:** 2026-09-24

**Authors:** Jaejun Jeong, Min Kyun Kang

**Affiliations:** ^1^ Department of Thoracic and Cardiovascular Surgery, Haeundae Paik Hospital, Inje University College of Medicine, Busan, Republic of Korea, inje.ac.kr

**Keywords:** case report, elderly patient, pleurodesis, pneumonitis, *Viscum album*

## Abstract

Secondary spontaneous pneumothorax in elderly patients is often challenging to treat surgically; hence, conservative management strategies, such as chemical pleurodesis, are frequently utilized. *Viscum album* extract is an established sclerosing agent used for pleurodesis, though some studies have reported associated complications, including acute pneumonitis. Here, we report the case of a 93‐year‐old male with secondary spontaneous pneumothorax who developed acute chemical pneumonitis following pleurodesis with *V. album* extract. Despite the initial radiologic severity, the patient was successfully managed and exhibited a benign clinical course.

## 1. Introduction

Secondary spontaneous pneumothorax in elderly patients is frequently difficult to manage surgically because of age‐related frailties and comorbidities, making conservative approaches like pleurodesis the preferred therapeutic choice. *Viscum album* extract is a plant‐derived chemical agent widely recognized for its efficacy in pleurodesis, particularly for malignant pleural effusions [1, 2]. Recent literature has also documented its application in benign conditions such as persistent air leaks and pneumothorax, alongside its potential adverse effects, including acute pneumonitis [3]. Generally, acute pneumonitis following chemical pleurodesis can be severe or even fatal in very elderly populations.

In this report, we present a rare case of a 93‐year‐old patient with secondary spontaneous pneumothorax who developed acute pneumonitis after undergoing bedside pleurodesis with *V. album* extract but achieved a full recovery through an exceptionally benign clinical course.

## 2. Case Presentation

**Figure 1:**
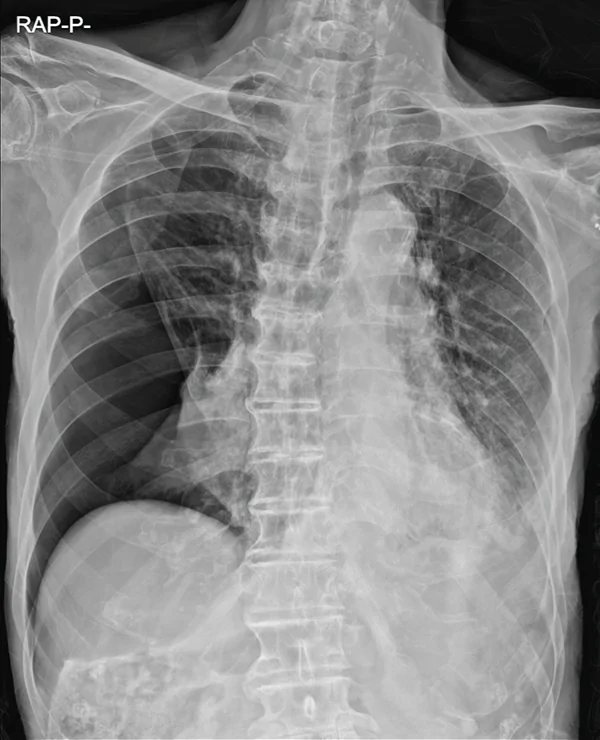
Initial chest radiograph demonstrating a large right‐sided spontaneous pneumothorax.

A 93‐year‐old man presented to our emergency department with the sudden onset of chest discomfort and dyspnea. He had no other active medical conditions under treatment. Upon physical examination, breath sounds were markedly diminished over the right hemithorax. A chest radiograph revealed a large pneumothorax in the right pleural cavity (Figure 1). A review of a chest computed tomography scan obtained 3 years prior demonstrated severe, bilateral panlobular emphysema.

Following the immediate insertion of a chest tube, a persistent air leak was observed for more than 5 days. Given the patient′s advanced age and poor underlying lung parenchyma, chemical pleurodesis was pursued rather than surgical intervention. Local anesthesia was first achieved by instilling 2% lidocaine through the chest tube. Subsequently, three ampoules of *V. album* extract (ABNOVA Viscum F) mixed with 50 mL of normal saline were injected. The patient was instructed to change positions (supine, prone, and left/right lateral) at 30‐min intervals over 2 h to ensure uniform distribution of the agent across the entire pleural surface. To prevent premature evacuation of the solution, the chest tube drainage line was clamped and suspended approximately 60 cm above the patient.

**Figure 2:**
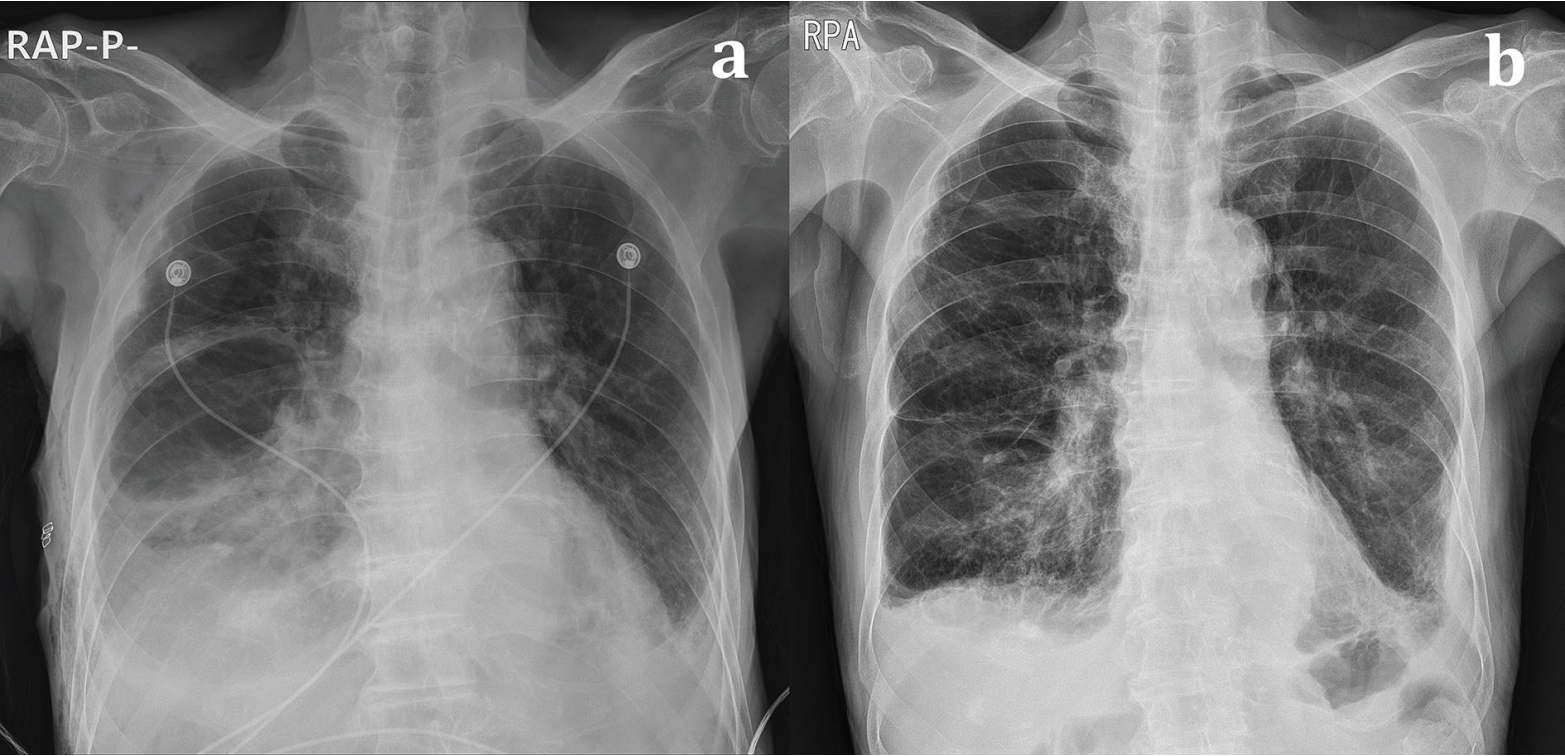
(a) Chest radiograph obtained on postpleurodesis Day 3 showing increased pleural effusion and dense infiltration in the right lower lung field, highly suggestive of acute pneumonitis. (b) Follow‐up chest radiograph showing near‐complete resolution of the effusion and infiltrates after conservative management.

On the day following the procedure, the air leak had completely resolved, and the chest tube was successfully removed. However, on postprocedural Day 3, the patient complained of worsening dyspnea. A follow‐up chest radiograph demonstrated an increase in pleural effusion accompanied by new infiltrates in the right lower lung field (Figure 2a). Laboratory findings revealed a markedly elevated C‐reactive protein (CRP) level of 19.62 mg/dL and a white blood cell (WBC) count of 10.09 × 10^9^/L. Based on the focal consolidation and effusion confined to the ipsilateral lung immediately following the procedure, combined with the rapid spike in inflammatory markers, the patient was diagnosed with acute chemical pneumonitis, potentially exacerbated by localized extrapleural deposition of the agent.

Supportive care was immediately initiated, consisting of supplemental oxygen, mucolytics, and empirical broad‐spectrum antibiotics to cover potential secondary infection. By Day 5 of supportive therapy, his respiratory symptoms had significantly improved, and subsequent chest radiographs showed a substantial reduction in both the pleural effusion and the parenchymal infiltrates (Figure 2b). The patient was discharged in stable condition on the 16th hospital day.

## 3. Discussion

Although surgical intervention remains the definitive treatment for primary and recurrent spontaneous pneumothorax, chemical pleurodesis serves as a crucial alternative for patients who cannot tolerate general anesthesia or major thoracic surgery. Conventional sclerosing agents include erythromycin, tetracycline, and talc slurry [4–7]. In this case, *V. album* extract was deliberately selected over talc slurry due to distinct clinical and practical imperatives. First, the patient was managed with a small‐bore chest tube (12 Fr), which posed a substantial risk of incomplete drainage or tube occlusion if a viscous talc slurry had been introduced. Second, *V. album* extract offered a more cost‐effective alternative to relatively expensive medical‐grade talc within our local healthcare setting. Finally, this choice was guided by recent clinical data demonstrating the noninferior efficacy and highly favorable safety profile of *V. album* extract for pleurodesis. Consequently, we determined that *V. album* extract represented the safest and most logistically viable option for this vulnerable patient.

Common complications associated with chemical pleurodesis include pleuritic chest pain, transient fever, and less frequently, acute pneumonitis [8–11]. Although autologous blood patch pleurodesis and hypertonic glucose solutions are occasionally utilized due to their simplicity and low toxicity profiles, the current evidence backing them remains limited to small, retrospective cohorts [12].

Differentiating between chemical pneumonitis and bacterial pneumonia is a critical clinical challenge, as they dictate divergent management pathways. Although chemical pneumonitis involves a sterile, hyperacute inflammatory response that typically manifests within hours of chemical exposure, bacterial pneumonia represents an infectious process stemming from parenchymal pathogen proliferation, usually unfolding over several days. Although CRP levels rise sharply in both conditions, patients in the hyperacute phase of chemical pneumonitis frequently present with a normal or only marginally elevated systemic WBC count [13].

In very elderly individuals, acute pulmonary inflammation can easily transition into fatal acute respiratory distress syndrome (ARDS). However, accumulating regional evidence suggests that acute pneumonitis induced by *V. album* pleurodesis often follows a uniquely benign clinical course, resolving much faster and more completely than what the initial, alarming chest radiographs might imply [14, 15].

In conclusion, this case illustrates that although secondary spontaneous pneumothorax in nonsurgical elderly patients can be effectively managed with *V. album* extract pleurodesis, clinicians must remain vigilant regarding postprocedural chemical pneumonitis. Fortunately, this specific complication appears to carry a benign prognosis and can be successfully navigated with timely supportive care alone. Therefore, chemical pleurodesis using *V. album* extract remains a highly viable therapeutic option even in ultraelderly or heavily comorbid populations.

## Author Contributions

Jaejun Jeong was involved in investigation, writing—original draft, and writing—review. Min Kyun Kang was involved in editing and final approval of the manuscript.

## Funding

No funding was received for this manuscript.

## Disclosure

Jaejun Jeong and Min Kyun Kang approved the manuscript.

## Consent

The authors declare that appropriate written informed consent was obtained for the publication of this manuscript and accompanying images.

## Conflicts of Interest

The authors declare no conflicts of interest.

## Data Availability

The data that support the findings of this study are available on request from the corresponding author. The data are not publicly available due to privacy or ethical restrictions.
